# ATP hydrolytic activity of purified Spf1p correlate with micellar lipid fluidity and is dependent on conserved residues in transmembrane helix M1

**DOI:** 10.1371/journal.pone.0274908

**Published:** 2022-10-20

**Authors:** Johan Ørskov Ipsen, Danny Mollerup Sørensen

**Affiliations:** 1 Center for Membrane Pumps in Cells and Disease—PUMPKIN, Danish National Research Foundation, Copenhagen, Denmark; 2 Department of Plant and Environmental Sciences, University of Copenhagen, Frederiksberg C, Denmark; 3 Department of Geoscience and Natural Resource Management, University of Copenhagen, Frederiksberg C, Denmark; University of Pittsburgh, UNITED STATES

## Abstract

P5A ATPases are expressed in the endoplasmic reticulum (ER) of all eukaryotic cells, and their disruption results in pleiotropic phenotypes related to severe ER stress. They were recently proposed to function in peptide translocation although their specificity have yet to be confirmed in reconstituted assays using the purified enzyme. A general theme for P-type ATPases is that binding and transport of substrates is coupled to hydrolysis of ATP in a conserved allosteric mechanism, however several independent reports have shown purified Spf1p to display intrinsic spontaneous ATP hydrolytic activity after purification. It has never been determined to what extend this spontaneous activity is caused by uncoupling of the enzyme. In this work we have purified a functional tagged version of the *Saccharomyces cerevisiae* P5A ATPase Spf1p and have observed that the intrinsic ATP hydrolytic activity of the purified and re-lipidated protein can be stimulated by specific detergents (C12E8, C12E10 and Tween20) in mixed lipid/detergent micelles in the absence of any apparent substrate. We further show that this increase in activity correlate with the reaction temperature and the anisotropic state of the mixed lipid/detergent micelles and further that this correlation relies on three highly conserved phenylalanine residues in M1. This suggests that at least part of the intrinsic ATP hydrolytic activity is allosterically coupled to movements in the TM domain in the purified preparations. It is suggested that free movement of the M1 helix represent an energetic constraint on catalysis and that this constraint likely is lost in the purified preparations resulting in protein with intrinsic spontaneous ATP hydrolytic activity. Removal of the N-terminal part of the protein apparently removes this activity.

## Introduction

P-type ATPases are active membrane pumps that are driven by ATP-dependent autophosphorylation of a conserved aspartate residue during their catalytic cycle. They have a well characterized reaction mechanism in which transport binding and release is coupled to formation and degradation of this transiently phosphorylated intermediate. P1-P3 type ATPases are cation transporters whereas P4 ATPases transport lipids [1]. The activity of P5-type ATPases was for a long time obscure but they were recently shown to transport small molecule species [2]. The human genome contains 5 genes that encodes for P5 type ATPases. ATP13A1 represents clade P5A, which is highly conserved between fungi and animals with one member in each investigated species. ATP13A2, ATP13A3, ATP13A4 and ATP13A5 belong to clade P5B and diversified from one isoform in fungi and primitive animals to a maximum of four in mammals by successive gene duplication events in vertebrate evolution [3]. Loss of P5-type ATPase function is related to severe disease conditions in humans [4–8].

The model eukaryote *Saccharomyces cerevisiae* contain only two P5-ATPases, the P5A ATPase Spf1p and the P5B ATPase Ypk9p. Spf1p is localized in ER membranes and its genetic deletion causes a pleiotropic phenotype characterized by ER stress, glycoprotein processing defects, abnormal protein targeting, alterations in lipid and sterol content and distribution, and the loss of Ca^2+^ and Mn^2+^ homeostasis (reviewed in [9]). Although Spf1p appears to display intrinsic ATP hydrolysis after purification this activity has not yet been linked to transport of any substrate(s). Several reports have shown that ATP hydrolytic activity of the enzyme is comparable to other low activity transport ATPases after purification [10–12]. So far, no dependence of the intrinsic activity towards cations have been shown and in fact all data so far show a loss of intrinsic activity at higher concentration, indicating that P5 ATPases are not cation pumps. Phosphatidylinositol-4-phosphate (PI4P) have been established to positively effect ATP hydrolytic activity although the lipid is more likely to act as a regulator than a transport substrate [12].

Recently the P5A subclass have been proposed to act as a transmembrane helix dislocase in the ER with the role of extracting mistargeted proteins from the endoplasmic reticulum [13]. This was based on findings that Spf1p bind to model tail anchored (TA) proteins and is needed for ATP-dependent dislocation of these in *in vitro* assays. More rigorous biochemical evidence for its involvement in this process using the purified enzyme in reconstituted assays is still missing and substrate specificity remains to be studied in relation to ATP hydrolytic activity. The poly-alanine peptide sequence that could be modelled into the binding site in the Cryo-EM structure so far seems to be the most profound candidate in this respect [13]. However, the P5A-type ATPase CATP-8 in *C*. *elegans* have also been found to remove ectopically located mitochondrial tail-anchored (TA) and signal-anchored (SA) proteins from the ER membrane [14], act as a key regulator controlling translocation of the DMA-1 receptor to the ER [15] and is required for translocation of EGL-20/Wnt polypeptide in neuronal migration [16] which lend further credit to the model that P5A ATPases could act as polypeptide translocators.

The human P5B ATPase, ATP13A2, was recently established as a polyamine transporter needed for cellular uptake and lysosomal secretion of labelled polyamines and with a polyamine stimulated ATPase activity that coincides with transport in reconstituted assays [2]. Likewise, polyamine stimulated activity was later shown for human ATP13A3 [17] and for yeast Ypk9 [18].

In this work we have tested the effect of different detergents on the spontaneous ATP hydrolytic activity of purified Spf1p reactivated in mixed lipid/detergent micelles. We identify polyethylene glycol containing detergents (Tween20, C12E8 and C12E10) to induce highest activity using a yeast polar lipid matrix and that the increase in activity correlates to the degree of fluidity measured in the lipid/detergent micelles by Laurdan fluorescence. We further show that mutation of conserved residues in M1 disrupts this effect and that an intact N-terminal covering the two P5A specific transmembrane helixes (Ma and Mb) are required for this functionality of the purified enzyme. It is speculated that the spontaneous nature of ATPase activity observed so far could be related to a switch in the enzyme that allosterically responds to physicochemical changes in the environment surrounding the transmembrane part of the protein and that purification of the membrane bound protein therefore results in preparations that display spontaneous ATP hydrolysis.

## Materials and methods

### Expression and purification of Spf1p

Expression and purification were performed as described in [12]. *spf1* deletant cells in the BY4741 background (Mat:α; his3D1;leu2D0;lys2D0;ura3D0;SPF1::kanMX5) were transformed by the lithium acetate method generating transformants that expresses Spf1p or Spf1p constructs carrying the following mutations: Spf1p-D487A; Spf1-E310A; Spf1p-F202A,F204A,F207A; Spf1p-F202H,F204H,F207H. Mutations were performed by PCR mutagenesis and confirmed by sequencing. The expression plasmids are 2μ episomal plasmids with histidine selection marker, containing the SPF1 gene controlled by a galactose inducible promotor and with a fusion sequence encoding a C-terminal FLAG-RGS10His. SDS-PAGE and western blot analysis were performed as standard procedures described in [12]. In-gel sample preparation was performed as previously described [19]. Mass spectrometry analysis was performed using a Thermo Fisher Orbitrap Exploris 480 system coupled to an EvoSep One nano-liquid chromatography system. Briefly, a data-dependent acquisition method was utilized on the Orbitrap Exploris 480 system running in positive mode. A top10 method with an MS1 resolution of 120,000 and the MS2 (MS/MS) resolution set to 30,000 was utilized. A 30 samples-per-day (44 minutes per sample) reversed-phase method on the EvoSep was utilized to separate peptides extracted from in-gel trypsin digestion. The resulting data was searched against the UniProt Saccharomyces cerevisiae fasta database using the MaxQuant software. A false discovery rate of 1% was used at the peptide and protein level. Intensity based absolute quantitation (iBAQ) values generated from MaxQuant analysis were utilized to determine proteome composition of the protein bands.

#### Complementation in spf1 deletant cells

Yeast cells were grown in YPD (1% wt/vol yeast extract, 2% wt/vol bactopeptone, 2% wt/vol glucose) or YPG (1% wt/vol yeast extract, 2% wt/vol bactopeptone, 2% wt/vol galactose) medium. Selection was performed on a synthetic defined minimal medium (0.7% wt/vol yeast nitrogen base, 0.02 mg/ml amino acid, 50 mM succinic acid-Tris, pH 5.5) containing the appropriate dropout nutritional supplements (Sigma) and 2% wt/vol glucose (SD) or galactose (SG). For plates, 2% wt/vol agar was added. For complementation tests, wild-type BY4741 yeast and transformed spf1 cells were grown overnight on YPD and selective SD plates, respectively, suspended in sterile ultrapure H2O, and diluted to OD600 = 1. A dilution series of OD600 = 1, 0.1, and 0.01 was made with sterile H2O, and 5 μl of each dilution was dropped onto YPD or YPG plates with or without inhibitors at the indicated concentrations. Spotted cells were grown for 2 d at 30°C.

*Detergent and lipids*. Lipid reactivation of purified Spf1p was performed essentially as described in [12]. Yeast polar lipid extracts (Avanti 190001) were dissolved in chloroform from the vial provided by the manufacturer and 1mg fractions were aliquoted into glass vials and stored at -20⁰C under N_2_. 244,7μL Reconstitution buffer (50 mM Tris-HCl pH 7.2, 50 mM NaCl, 0.5% wt/vol indicated detergent) was used to resuspend the lipids, resulting in a lipid concentration of 4.086 mg/mL. Resuspended lipid/detergents mixtures were homogenized using ultra-sonification in a water bath for at least 5 minutes. The detergents used where: Tween 20 (Polysorbate 20, Merck 817072), n-Octyl-β-D-Glucopyranoside (Anatrace O311-1), Deoxy Cholate (Sigma D6750), Fos-choline-13 (Anatrace F310S-1), Big CHAPS (Anatrace B310-1), Polyoxyethylene(8)dodecyl Ether (C12E8, Anatrace APO128), (Polyoxyethylene(9)dodecyl Ether (C12E9, Anatrace APO129) and Polyoxyethylene(10)dodecyl Ether (C12E10, Anatrace AP1210).

*ATPase measurements*. Measurement of ATPase activity was performed essentially as described in [12]. Prior to the assay Spf1p was reactivated with the solution containing lipid and detergents described above to a defined amount of detergent determined as w/w % of the protein content. The lipid to protein ratio was 3,552 mg lipid per mg protein and 5 μg of Spf1p was used for each replicate. The mix was incubated on ice for 30 minutes prior to assay and the assay was run at the indicated temperature in a Grant Qbd4 heater for 30 minutes and stopped by the addition of ice-cold 300 μL stop buffer containing 0,154 M Ascorbicacid (AppliChem A1052), 0,55 mM Ammonium-heptamolybdate (Merck 1.01180) and 1 w/w% SDS (Sigma L3771). The assay mixture was kept on ice for 15 minutes and of 450 μl 2% glacial acetic acid (Merck 100063), 2% trisodium citrate (Sigma C8532) and 2% sodium arsenite (Sigma S7400) was added to the mixture. This was left on tabletop for 1 h at room temperature and absorbance at 860 nM was measured with a spectrophotometer (Thermo Genesys 10 Bio) using a water sample and concentration of phosphate determined based on linear regression to a standard curve. All determinations were carried out as technical triplicates of two biological replicates (*n* = 6). For experiments at different temperatures the reaction buffer was heated to the temperature the assay would be performed at and the pH was adjusted at the elevated temperature.

*Laurdan fluorescence measurements*. Measurements were performed on mixed YPL/detergent micelles containing purified Spf1p that was used for ATPase measurements using a Laurdan to YPL ratio of 1:1000 (w/w). A dry film was made in glass vials from a CHCl_3_/MeOH (1/1) solution containing 1mg YPL and 1μg Laurdan. The film was solubilized with 1.25ml buffer Buffer A (MOPS pH7.4 50mM, KCl 50mM) and then sonicated 5 min in a water bath. The assay was performed in 96 microwell plates and assayed on a Multi-Detection Microplate Reader (Biotek H2 Synergy). 50 μl detergent in water (1%) was added to each well to reach desired concentration in final 100ul vol. and a dilution series in 1:1 ratio with H_2_O so that all wells contain 50ul detergent mix with varying concentration. 25μl buffer A and 25μl YPL/Laurdan mix to each well to start the assay. Fixed excitation at 350nm was used and emission data was collected in 5nm incremental steps between 380 and 520 nm. General Polarization was calculated as GP = (*I*_435_- *I*_500_) / (*I*_435_+ *I*_500_) where *I* = measured intensity. Measurement was performed at 35⁰C. Background control and emission maxima were verified in the individual datasets containing the 261 datapoints covering all emission wavelengths in each experiment. Data is reported as the average of three biological replicates with error bars indicating the standard error.

*Bioinformatics*, *alignments and modelling*. Alignments were performed as described in [20], Calculation of free energy for TM helix prediction was performed using the TOPCONS prediction algorithm [21]. Modelling of Spf1p was performed using the SWISS-MODEL homology modeler [22] based on a modified sequence of the Ca^2+^ ATPase (ATP2A1: P04191) in which the residues in M1, 2 and 4 were switched to the those found in Spf1p. The modelled structure is comparable to the structure that was later reported [13].

## Results

### Ethylene-glycol containing detergents increase ATPase activity of purified Spf1p in mixed lipid/detergent micelles

Spf1p was expressed and purified using a protocol that was previously optimized to remove contaminants that can interfere with Spf1p activity [12, 23]. The purified preparations were confirmed to contain mainly Spf1p protein as determined by amino acid analysis and mass spectrometry, although in some of the produced fractions we could identify glyderaldehyde-3-phosphate dehydrogenase as a minor contaminant (S1 Fig).

**Fig 1:**
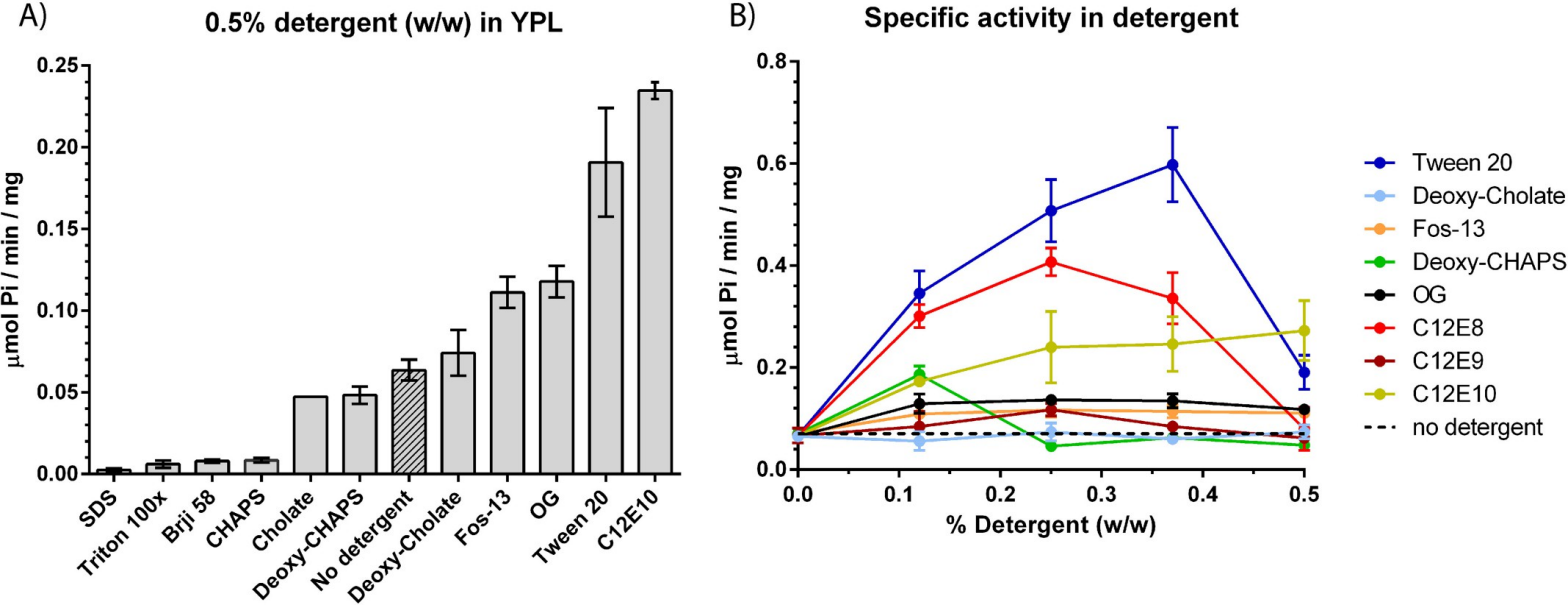
Detergent screen on purified Spf1p in yeast polar lipid (YPL). A) Specific activity in μmol Pi/min/mg when indicated detergents are included at 0.5% detergent (w/w) in the mixed lipid/detergent micelles. B) Specific activity in μmol Pi/min/mg when detergents are titrated into the lipid micelles between 0 and 0.5% detergent (w/w). Detergents that showed a detrimental effect on activity (SDS, triton x100, Brij58 and CHAPS) is not shown. Baseline activity with no detergent is shown as dotted line. All experiments are performed as biological replicates and technical triplicates (*n* = 6) and is reported as the average value of these with error bars indicating the standard error (except cholate in 1A, *n* = 2 and only average value reported).

In order to analyze how detergents affect activity of Spf1p we performed a detergent screen on the purified protein residing in mixed lipid/detergent micelles. A yeast polar lipid matrix (YPL) which is a rich source of phosphatidylinositol-4-phosphate (PI4P) was used as the lipid source. During reactivation the lipid matrix was doped with detergents representing different classes of physicochemical properties (ionic state, polarity and size of hydrophic moiety). Detergents were initially screened at 0.5% (w/w) (Fig 1). This initial screen allowed us to identify individual detergents that showed either strong negative or positive effects on activity of the re-lipidated protein as compared to addition of lipids only. Here Tween20 and C12E10 showed the strongest stimulatory effect on ATP hydrolysis, with both detergents increasing ATPase activity to above ~0.2 μmol Pi/min/mg which is ~2x fold of what was previously reported using n-Octyl-β-D-Glucopyranoside (OG) in a pure POPC/PI4P matrix (Fig 1A, [12]).

To extend the analysis we proceeded by analyzing individual detergents more closely by titration of the detergents into the mixed lipid/detergent micelles (Fig 1B). Here we observed that the effect of Tween20 could be further increased at lower concentrations resulting in an apparent ~10x fold increase in ATPase activity reaching a maximum of ~0.6 μmol Pi/min/mg at around 0.38% (w/w). C12E10 showed a maximal activity of ~0.22 μmol Pi/min/mg at 0.5% (w/w) and the shorter C12E8 showed a maximal activity of ~0.4 μmol Pi/min/mg at 0.25% (w/w). A shared feature of the detergents that increased ATPase activity of Spf1p is the presence of polyethylene glycol ethers in the hydrophilic part of the detergents. Tween20 is characterized by the presence of branched polyethylene glycol ethers while C12E8 and C12E10 both contains polyethylene mono glycol ethers. The stimulatory effect was absent for detergents that had no ethylene glycol moieties (Deoxy-Cholate, Deoxy-CHAPS, Octyl glucoside and Fos-13, Fig 1B).

We show that inclusion of the ethylene-glycol containing detergents (Tween20, C12E10 and C12E8) increase spontaneous ATPase activity of purified Spf1p in mixed polar lipid/detergent micelles with Tween20 inducing the largest increase in activity.

### Detergent induced activity is not the result of an altered state transition of Spf1p

**Fig 2:**
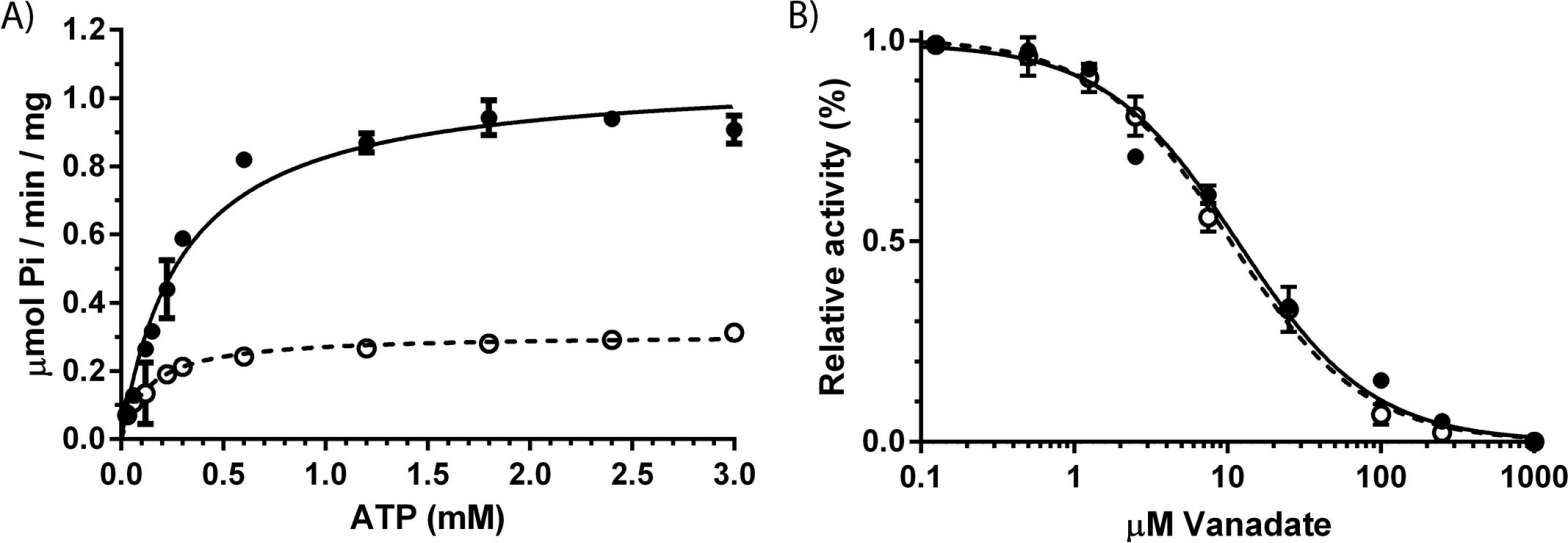
ATP affinity and vanadate sensitivity of purified Spf1p in mixed YPL/detergent micelles (0.35% w/w). A) ATP affinity of Spf1p in YPL/OG micelles (open circles, dotted line with *K*_m_ = 0.1332 ± 0.0189 mM ATP and *V*_*max*_ = 0.3065 ± 0.0969 μmol Pi/min/mg) and in YPL/Tween20 micelles (black circles, black line with *K*_m_ = 0.2977 ± 0.02569 mM ATP and *V*_*max*_ = 1.072 ± 0.0270 μmol Pi/min/mg). Curve fittings was performed using Michaelies-Menten kinetics. B) Vanadate sensitivity of Spf1p in mixed YPL/OG micelles (open circles, dotted line with *K*_i_ = 10.30 ± 0.7471 μM) and YPL/Tween20 (black circles, black line with *K*_i_ = 10.30 ± 0.7471 μM). Curve fittings was performed using Substrate inhibition kinetics. All experiments are performed as technical triplicates (*n* = 3) and is reported as the average value of these with error bars indicating the standard error.

P-type ATPases undergoes a cyclic reaction mechanism by oscillating between substrate bound and substrate free states (E1-E2 reaction mechanism), binding of ligands such as transport substrates, regulatory ligands and nucleotides can modify catalytic turnover of the cycle by binding to specific intermediate states [1]. A classical way to probe shifts in state transition of P-type ATPases is by measuring sensitivity towards vanadate which block enzymatic activity by acting as a phosphate leaving group mimic in the E2 state. To test if addition of detergents increased activity of Spf1p by introducing a shift in state equilibrium, we measured ATPase affinity and performed a vanadate sensitivity experiment on the enzyme prepared in YPL micelles containing either OG or Tween20 at 0.35% (w/w) (Fig 2). ATP affinity decreased in the presence of Tween20 while vanadate sensitivity was unaffected, which leads us to conclude that state equilibrium of Spf1p is similar in the two preparations and that Tween20 induced activity of Spf1p is not the result of an altered state transition of the enzyme.

### Tween20 and C12E8 increase fluidity of lipid/detergent micelles carrying Spf1p

Temperature, lipid composition and detergents affect fluidity and disordering of lipid phases. A commonly applied approach to measure these effects is by measuring the fluorescent properties of intercalating probes which respond to the dipole changes in water molecules close to surface of the lipid environment. One such molecule is Laurdan (6-dodecanoyl-2-dimethylaminonaphthalene). Its fluorescence is usually expressed in terms of a “generalized polarization” (GP) parameter, that is calculated by the fluorescence intensities at two predetermined wavelengths (435 nm and 500 nm) where GP varies between 1 (no solvent effects) and -1 (complete exposure to bulk water). Since the presence and mobility of water molecules depends on the mobility of lipid molecules in the lipid phase, the GP value of Laurdan can be used to report changes in lipid phase transitions from ordered to more disordered phases [24–26].

**Fig 3:**
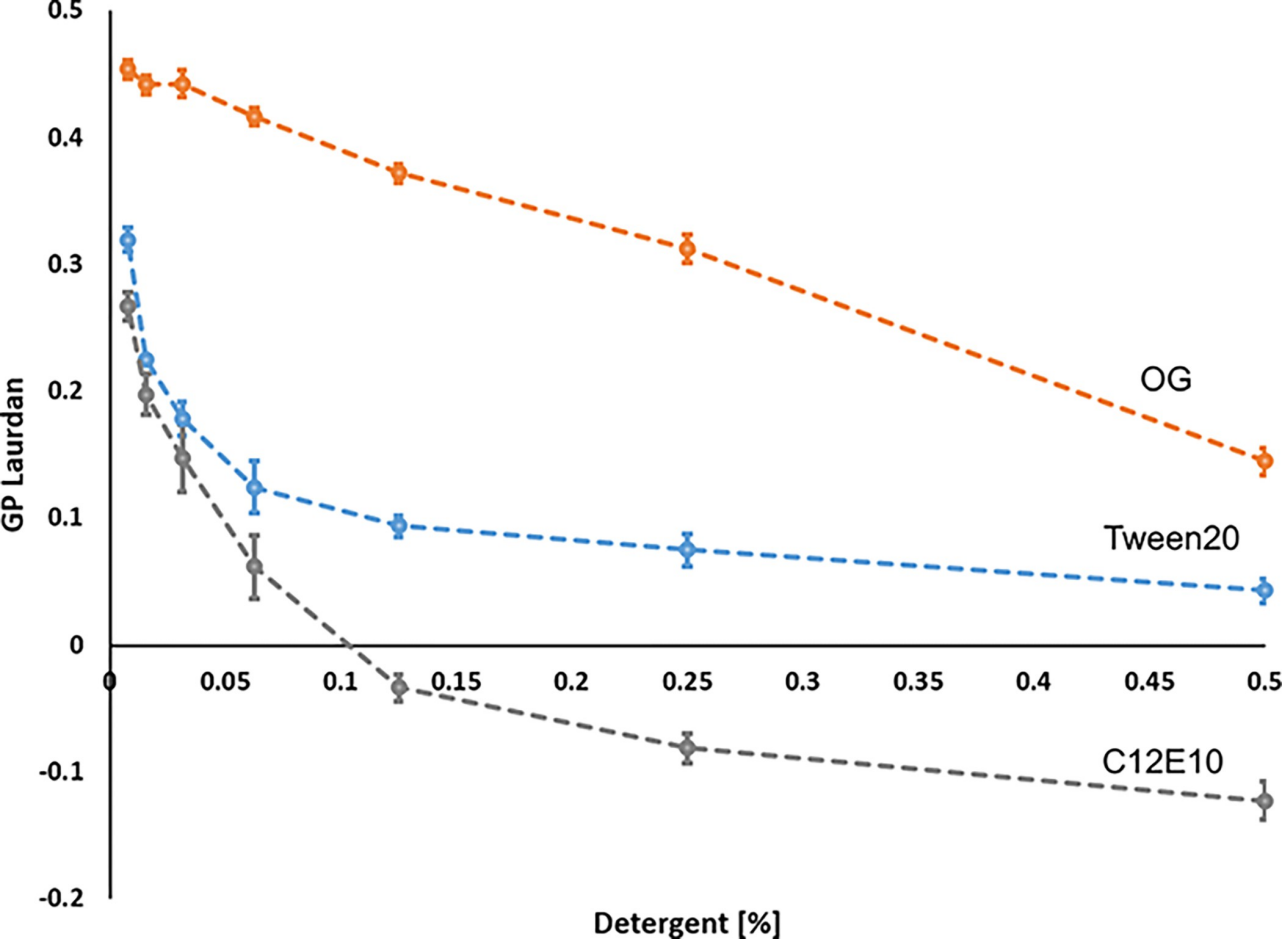
Laurdan fluorescence experiment on mixed YPL/detergent micelles containing Spf1p at different concentration of detergent (0–0.5% w/w) and temperature (25–40°C). Generalized Polarization was calculated based on the fluorescent intensities of Laurdan using the equation GP = (*I*435nm−*I*_500nm_) / (*I*_435nm_+ *I*_500nm_), where *I* = measured intensity. Decreasing GP values indicate increasing lipid phase disorder. Different detergents were used in the mixed lipid/detergent micelles; OG (orange), C12E8 (grey) and Tween20 (blue). Data is shown as the average of 3 biological replicates with error bars indicating the standard error.

When analyzing Laurdan fluorescence of mixed lipid/detergent micelles carrying Spf1p protein we observed that Tween20 and C12E8, were able to sharply increase lipid phase disorder as indicated by a decrease in the GP value of Laurdan. In contrast lipid phase disorder for OG was much less pronounced (Fig 3). Moreover, this followed an inverse relationship with the ATP hydrolytic activity. Addition of C12E8 result in a rapid decrease in Laurdan GP which stabilized at around 0.125–0.25% (w/w). The ATP hydrolytic activity of Spf1p at the same conditions also show an optimum at around 0.25% (w/w) (refer to Fig 1B). Increased lipid phase disorder hereby correlates with Spf1p ATP hydrolytic activity. A similar relationship between lipid phase disorder and activity was also observed for Tween20 –i.e. a steady decrease in Laurdan GP value up to 0.25% (w/w) which correlates with an increase in ATP hydrolytic activity up to 0.4% (w/w) detergent. In contrast, OG resulted in a much less pronounced change in lipid phase anisotropy as indicated the overall stable GP value of Laurdan, which also coincides with an overall stable ATP hydrolytic activity at all tested detergent concentrations (Figs 1 and 3).

The data hereby suggest that the effect of Tween20 and C12E8 on Spf1p activity is correlated to the concentration at which these detergents to increase fluidity of the lipid environment.

### N-terminal domain is required for functionality of purified Spf1p

**Fig 4:**
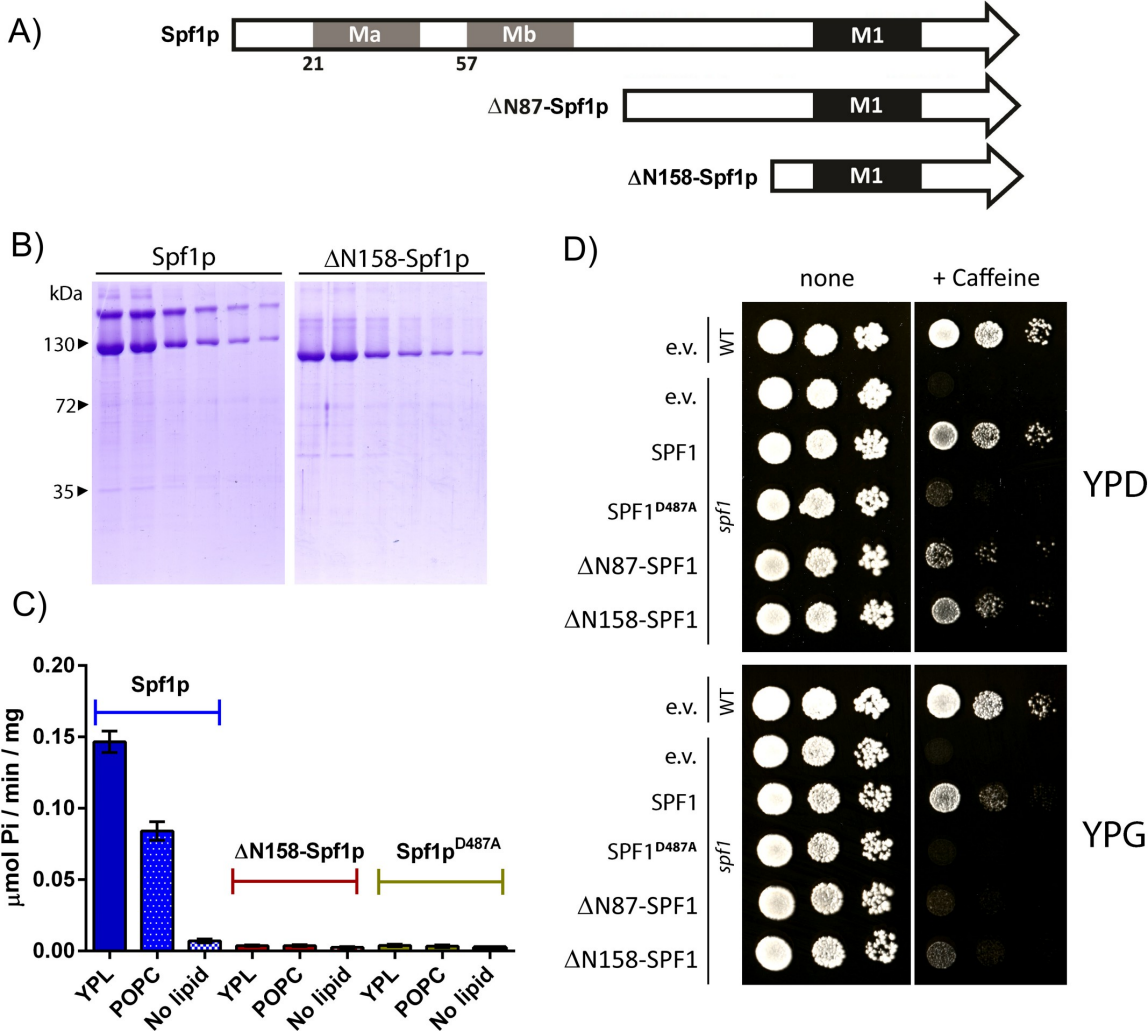
Viability of Spf1p and N-terminally truncated Spf1p. A) Schematic overview of N-terminal truncations performed by genetically removing the genetic sequence that codes for the first 87 and 157 amino acids of the expression construct. Ma, Mb and TM1 is indicated in accordance with [3, 20]. B) SDS-PAGE showing the eluates for the purified fractions of the full-length expression construct (Spf1p) and the N-terminally truncated expression construct (ΔN187-Spf1p). C) ATPase activity measured for full-length (Spf1p), N-terminally truncated expression construct (ΔN187-Spf1p) and a dead control (Spf1p-D487A) after re-lipidation in mixed lipid/detergent micelles using OG as detergent (YPL, POPC) or with no lipids added. D) Complementation in *spf1* deletant cells using the same expression constructs. The catalytic dead mutation D487A and the empty vector is used as a control. Complementation was tested on 10 mM caffeine plates as described in [12]. Plates grown on glucose (YPD) repression of the promotor allows survival of cells expressing the N-terminally truncated version of SPF1, but high expression conditions on galactose (YPG) hinders survival.

When analyzing the purified fractions on SDS-PAGE gels, full length Spf1p was found to migrate as a double band with one band at the expected molecular size and one at approximately twice the expected size (S1 Fig), suggesting persistent dimerization of the protein even under strongly denaturizing conditions. The N-terminal part of Spf1p contain a signature domain with P5A specific transmembrane helixes (Ma and Mb) preceding the M1 transmembrane helix found in other P-type ATPases [20]. We therefore proceed to remove the first 158 amino acids from the N-terminal of the expression construct and purified the resulting protein (ΔN158-Spf1p). This truncation caused the protein to run as a single band, suggesting that the dimerization happens in this region (Fig 4). ΔN158-Spf1p further failed to show activation of ATPase activity when reactivated in mixed lipid/detergent micelles. The expression construct encoding ΔN158-Spf1p and a similar expression construct encoding another N-terminally truncated protein (ΔN87-Spf1p) were also both not able to show cellular rescue in genetic complementation experiments when high expression was induced with galactose, although the same constructs allowed survival during glucose repression of the promotor. These data indicate that the N-terminal part of Spf1p is required for functionality of the purified enzyme produced during high expression conditions.

### Integrity of the α-helical nature of M1 is important for functionality of purified Spf1p

**Fig 5:**
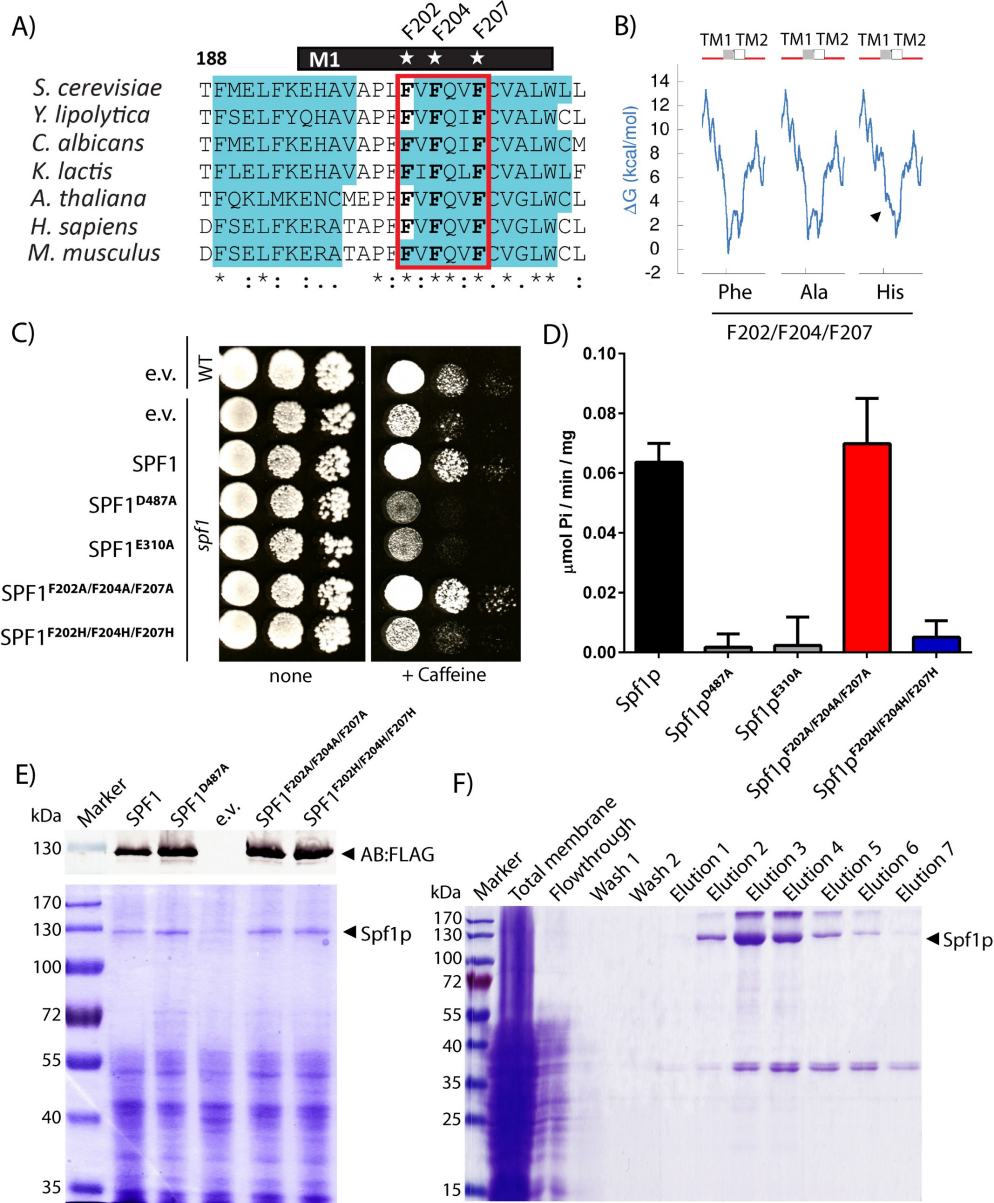
Viability of Spf1p and M1 mutated Spf1p. A) Alignment of M1 region of Spf1p and similar P5A ATPase sequences. Conserved residues F202, F204 and F207 indicated by star and predicted α-helical structure shown in blue. B) Calculated ΔG (kcal/mol) for folding of M1 and M2 in Spf1p and M1 mutated spf1p. Introduction of histidines, but not alanines, increase the calculated energetic requirement for proper folding into the α-helical structure. C) Complementation in *spf1* deletant cells using the expression constructs used in this study. The catalytic dead mutations D487A and E310A and the empty vector was used as a control. Complementation was tested on 10 mM caffeine plates as described in [12]. D) Specific activity of purified Spf1p mutants in mixed YPL/OG micelles. All experiments are performed as biological replicates and technical triplicates (*n* = 6) and is reported as the average value of these with error bars indicating the standard error. E) Expression analysis of the constructs used in this study, SDS-PAGE show expression of a ~130 kDa band corresponding to Spf1 and M1 mutated Spf1p. A western blot against the FLAG-tag using Anti-FLAG AB is shown for reference. F) SDS-PAGE gels of a typical purification showing relatively pure and homogenous preparations comparable to those presented in [12]. Fractions containing Spf1p were pooled for recovery of purified Spf1p.

P-type ATPases display a large degree of conformational rearrangement during transport with movements in the cytoplasmic domain being coupled to opening and closing of an access channel for transport substrates between M1, M2 and M3 in the TM domain. Especially M1 appears to display a large degree of movement where it alternates between being fully kinked in the substrate free state to being almost fully unwound in the substrate bound state [27]. The P5A subclass (including Spf1p) contain highly conserved residues in this region (M1 shown in Fig 4A, for region M2-M3 see [20]). In contrast to other model P-type ATPases like the Ca^2+^ ATPase (P2B class) most of these residues are hydrophobic in nature with phenylalanine being the most pronounced. A highly conserved proline is also identified in M1 of Spf1p (Fig 5A) which in other P-type ATPases acts as a helix breaking motif in the substrate free E2 form of the enzyme. *In situ* topology prediction suggests that partial removal of the hydrophobic character in the triple histidine mutant removes the ability of M1 to fold into a proper α-helix structure as indicated by the increased energetic requirement (ΔG) for folding (Fig 5B).

As the N-terminal part of Spf1p seemed to be important for function we proceeded to further test the effect altering M1 integrity by mutating the three conserved phenylalanines into either alanine or histidines. Although highly conserved, the three phenylalanines in M1 are not required for functionality, as indicated by the ability of a triple alanine mutated version of the enzyme (Spf1p-F202A, F204A,F207A) to sustain both ATP hydrolytic activity in YPL/OG micelles and to show cellular rescue in genetic complementation experiments. In contrast the histidine mutated version of the enzyme (Spf1p-F202H,F204H,F207H) display complete loss of both ATP hydrolysis and cellular rescue activity (Fig 5C and 5D) indicating that the hydrophobic nature of the sidechain residues is preferred over the more hydrophilic variants.

These data suggest that integrity of the helical nature of transmembrane helix M1 is important for the function of Spf1p although the role of conserved phenylalanine residues in this region is somewhat unclear.

### Three conserved phenylalanines in M1 are required for the Tween20 and C12E10 induced activity of Spf1p

**Fig 6:**
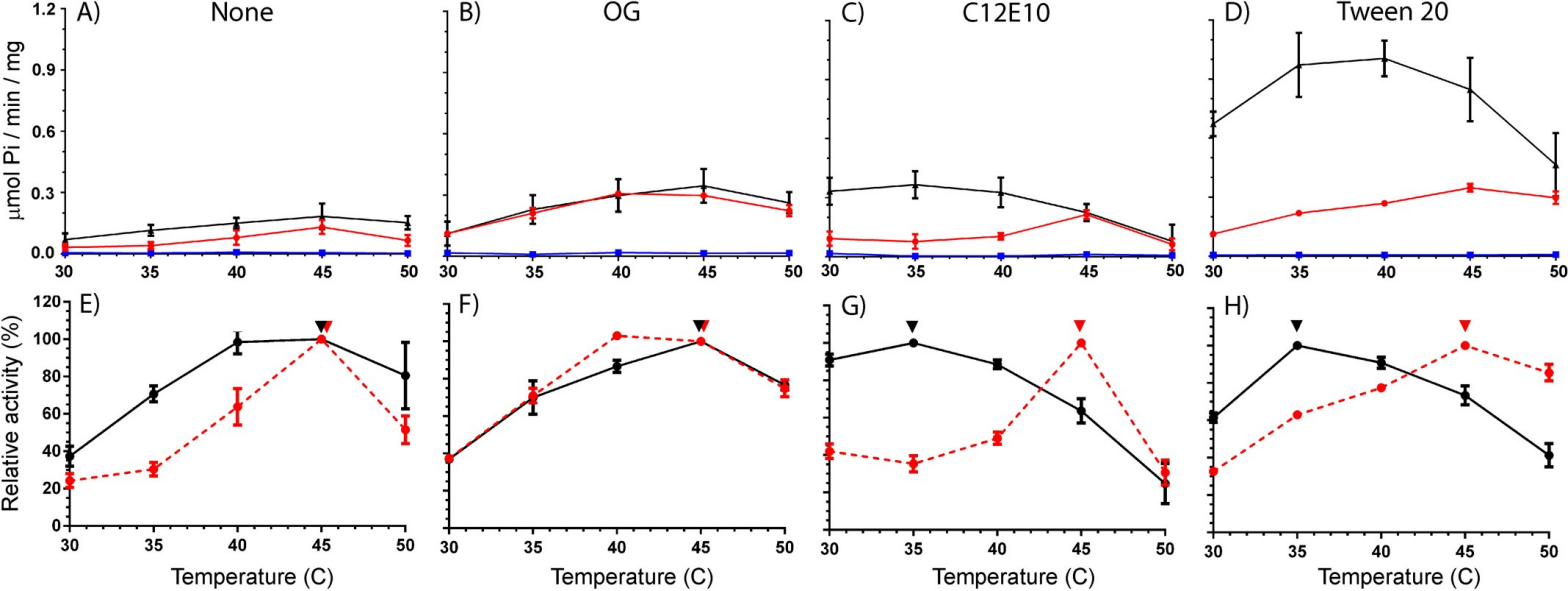
ATPase activity of Spf1p and M1 mutated Spf1p in mixed YPL/detergent micelles. A-D) Specific activity in μmol Pi/min/mg at indicated temperatures in absence of detergent (control) or in the presence of 0.35% (w/w) detergent as indicated. Spf1p (black), Spf1p-F202A,F204A,F207A (red) and Spf1p-F202H,F204H,F207H (blue). All experiments are performed as biological replicates and technical triplicates (*n* = 6) and is reported as the average value of these with error bars indicating the standard error (except cholate in 1A, *n* = 2 and only average value reported). E-H) The same data plotted as relative activities in relation to the maximal activity observed. Spf1p (black), Spf1p-F202A,F204A,F207A (red), Arrows indicate optimal temperature and error bars indicate the standard error.

The specific response to detergents that influence lipid disordering and an apparent high optimum temperature of Spf1p ATP hydrolytic activity at ~45⁰C [12] prompted us to further investigate the effects of mutating residues in the M1 region. As a control, measurements were performed in the absence of any added detergent and at fixed concentrations of either Tween20, C12E10 or OG where each detergent had stable optimal activity. The resulting data can be plotted as both specific activities (Fig 6A–6D) and as relative activities as percent of maxima in the temperature response plots for each experiment, which more clearly show the difference across the temperature span (Fig 6E–6H).

In confirmation of previous studies purified Spf1p display a temperature optimum at 45°C when reactivated with OG (Fig 5, specific activity ~0.37 μmol Pi/min/mg). A similar temperature response curve with optimum at 45°C is found in the absence of added detergent although at these conditions the enzyme has a lower activity (Fig 5, specific activity ~ 0.14 μmol Pi/min/mg). Addition of detergent hereby seems to be required for activation of some latent activity of Spf1p in the YPL matrix but otherwise OG appears to have no effect on the temperature response curve of the enzyme. The triple alanine mutated enzyme (Spf1p-F202A,F204A,F207A) behaves similarly to Spf1p, both in the absence of detergent and in the presence of OG with comparable specific activities and temperature response curves, while the triple histidine mutated control (Spf1p-F202H,F204H,F207H) displays complete loss of activity at all conditions suggesting that disruption of α-helix formation in M1 result in complete loss of activity.

In contrast, reactivation of Spf1p with C12E10 or Tween20 result in a dramatic effect on both the maximal specific activity and the temperature response. Both detergents increase specific activity in the physiological range around 30–35⁰C raising it either 4-fold for C12E10 (Fig 6, from ~0.10 to ~0.37 μmol Pi/min/mg) or 10-fold for Tween20 (Fig 5, from ~0.1 to ~1.05 μmol Pi/min/mg) when compared to the enzyme in the absence of added detergent. This response is associated with a shift in the temperature response curve which reaches an optimal temperature already at 35°C as compared to 45°C for the enzyme activated with OG or in the absence of detergent. The optimum temperature furthermore appears to be comparable between Tween20 and C12E10 indicating that the effect relates to a common characteristic between the detergents which is not found in OG. Although the triple Spf1p-F202A,F204A,F207A mutated enzyme behaves similar to Spf1p when activated with OG, mutation of the conserved phenylalanines in M1 removes the effect of Tween20 and C12E10 in the triple alanine mutant. Spf1p-F202A,F204A,F207A hereby retains its temperature optimum at 45°C in the presence of both Tween20 and C12E8 and fail to show high activity as observed for Spf1p at 35°C at these conditions. The increased turnover observed at elevated temperature can hereby be removed by mutation of specific conserved hydrophobic residues in the M1 domain of the enzyme.

## Discussion

In this work we report the effect of detergents on spontaneous activity of purified Spf1p in lipid/detergent micelles. We show that the spontaneous ATP hydrolytic activity of Spf1p correlates to the degree of disordering in the lipid/detergent micelles and that the correlation depends on conserved hydrophobic residues in M1.

Different lipids and detergents have previously been used by different research groups to confer activity to purified Spf1p (summarized in Table 1). In the initial studies OG was used together with an *E*. *coli* lipid extract [10]. Later C12E10 has been used together with phosphatidylcholine [11, 28] and OG has been used together with POPC/PI4P lipid extracts [12]. In the current work we report the highest recorded activity so far for Spf1p when the enzyme is reactivated with Tween20 in a yeast polar lipid extract.

**Table 1:** Specific activities reported in nmol/min/mg for purified and lipid/detergent reactivated Spf1p with the conditions for individual experiments shown. Star indicate activity measurements using radiolabeled ATP^32^, C12E10: Polyoxyethylene(10)dodecyl Ether, OG: n-Octyl-β-D-Glucopyranoside, PC/POPC: phosphatidylcholine and n.r.: not reported. For comparison polyamine stimulated activity of lipid activated ATP13A2 is ~150 nmol/min/mg [2] and for Ypk9p ~1.000 nmol/min/mg [18] both at 37⁰C, phosphatidylserine stimulated activity of the lipid flippase ATP8A1 is ~100.000 nmol/min/mg at 37⁰C [36], the activated state of the Pma1p H^+^ ATPase is reported at ~12.000 nmol/min/mg at 30⁰C [37] and activity for SERCA and SPCA Ca^2+^ ATPases are reported at ~12.000 nmol/min/mg and ~2500 nmol/min/mg respectively in proteoliposomes [38].

nmol Pi min^-1^ mg^-1^	Detergent	Lipid	Nucleotide/ Mg^2+^	Temp.	Protein determination	Reference
36	C12E10	PC	30μM ATP* / 2mM Mg^2+^	28°C	Bradford / BSA	*[28]*
40	OG	PC	3mM ATP / 8mM Mg^2+^	30°C	Bradford / γ-globulin	*[12]*
120	OG	*E*.*coli* total lipid extract	50μM ATP* / 5mM Mg^2+^	n.r.	BCA / n.r.	*[10]*
120	OG	Yeast polar lipid extract	3mM ATP / 8mM Mg^2+^	30°C	Bradford / γ-globulin	*This study*
160	OG	POPC/PI4P	3mM ATP / 8mM Mg^2+^	45°C	Bradford / γ-globulin	*[12]*
450	C12E10	Yeast polar lipid extract	3mM ATP / 8mM Mg^2+^	35°C	Bradford / γ-globulin	*This study*
800	C12E10	PC	3mM ATP / 5mM Mg^2+^	37°C	Bradford / BSA	*[11]*
1000	Tween20	Yeast polar lipid extract	3mM ATP / 8mM Mg^2+^	35°C	Bradford / γ-globulin	*This study*

Although the purity of individual preparations needs to be considered, we believe that several general observations can be established based on these collected efforts. First of all, it appears that from a biological perspective, the ATP hydrolytic activity of Spf1p has a high temperature optimum (between 35–45°C depending on the detergent and lipids used). Secondly, activity appears to be highest in heterogenous lipids together with polyethylene glycol containing detergents (Tween20, C12E8), where the yeast polar lipid matrix gives the highest observed response. Thirdly, in all cases ATP hydrolytic activity appears to be spontaneous in the purified system and happens in the absence of any apparent transport substrate. As shown in this report spontaneous activity correlates with the anisotropic state of the lipid environment and this correlation relies on conserved residues in M1. This suggest that the allosteric coupling between the TM part of Spf1p and its catalytic domain is intact in our preparations, as in the case of an allosteric uncoupling, spontaneous activity would either be expected to be released completely or not at all (and not as observed in a graduating manner related to the state of the lipid environment) and would further not be dependent on residues in the TM domain. Fourth, the recorded specific activities of Spf1p in both this and previous reports is well within the magnitude of substrate stimulated activity observed for P5B-type ATPases of similar purity [2, 18], even when accounting for the lower purity of crude Spf1p fractions used in previous studies, and certainly below the extreme activity overserved for other P-type ATPases of comparable purity (Table 1).

Detergents that seem to work best for preserving activity of Spf1p contain polyethylene glycol ethers in their hydrophilic moiety (C12E10, C12E8) and preferentially in a branched structure (Tween20). Although we cannot provide a direct answer for why C9E10 show no increase in activity above the level found for OG and thus fails to reach the same effect seen by C12E8 and C12E10, we speculate that lipid/detergent phase transitions might be limiting for specific chain structures. In comparison if the carbon chain in the hydrophobic part of the detergent exceeds ~10–12 residues and contain branching methyl groups (tritonX100, Brij58) it is likely to be unfavorable for Spf1p activity as well. Detergents that are based on a bile acid structure (deoxy-CHAPS, cholate, deoxycholate) have little or no effects on activity of purified Spf1p, although the presence of a zwitterion in the hydrophilic moiety of the bile acid structure seems to be detrimental for activity as demonstrated by sensitivity to CHAPS. In comparison, the presence of a polar headgroup containing multiple -OH groups in deoxy-CHAPS, and a zwitterionic headgroup can be tolerated in the absence of a bile acid moiety as demonstrated by a slight increase in activity that is comparable between Fos13 and OG. Further studies might reveal more about how the physicochemical properties of these detergents affect the lipid/detergent environment which likely would relate to changes in the lipid/detergent micelles.

Studies on perturbation of POPC lipid bilayers by solid state H^2^-NMR and isothermal titration calorimetry have revealed that addition of the C12E8 class of detergents produce a general disordering at all levels of the lipid bilayer whereas addition of OG only affect the fatty acyl chains in the inner part of the hydrophobic bilayer [29]. The effect of OG on the lipid environment is hereby drastically different from that of C12E8/C12E10 which also fit with our observations that the two detergents affect fluidity of the lipid environment differently. Due to the long polyether ‘head-group’ and a short hydrophobic tail of these detergents, the position and orientation of the detergent molecules along the micellar surface plane is conceivably not as restricted as it is for the lipid molecules. This could allow for a more flexible amphipathic environment around the protein in the mixed lipid/detergent micelles, in which the lipid molecules can occupy a broader distribution along the surface without a significant energetic cost. Our observations that increased micellar fluidity is associated with a higher rate of spontaneous ATP hydrolysis by Spf1p hereby converge on the model that the TM part of the enzyme is allowed a higher degree of movement under these conditions.

So far ATP hydrolysis of Spf1p have in all cases been observed to be spontaneous. This could imply that activity can be regulated by imposing forces from the lipid environment in the ER membrane which enforces the transporter into a low activity state when activity is not needed. Likely this repression is lost during purification of the enzyme. If this is true, it would present a new form of autoregulation in P-type ATPases—i.e. that they can be turned on by local physicochemical changes in the lipid environment. This would also fit with the recent findings that P5A ATPases show interaction with TM spanning helixes which at a high local concentration would affect the local physicochemical properties of the ER membrane surrounding the enzyme [13]. Indeed, the interplay between catalytic movements of P-type ATPases and the lipid environment have previously been speculated to be of significance for their activity based on molecular modelling of their action in the lipid environment [30]. It is hereby not inconceivable that local energetic restraints could keep the spontaneous ATP consumption of Spf1p in check when it is not needed, and that local changes in the lipid environment induced by increased concentration of misfolded TM peptides or changes in membrane sterol content could activate the enzyme [12, 13], although further studies are needed to examine this hypothesis. It was previously observed that deletion of the SPF1 gene results in general dyshomeostasis of lipids and sterols, but it is still unclear if this is a compensatory mechanism to relive stress from loss of its function or a more direct causal outcome from its deletion [12, 31].

Spf1p contains a P5A specific domain in its N-terminal region that cover two transmembrane helices (Ma and Mb) [3, 20]. We show here that at least in the purified form, this domain is required for spontaneous ATPase activity and that upon removal of this region the enzyme represents a state that cannot be reactivated by re-lipidation or in mixed lipid/detergent micelles. At least in the purified state the enzyme shows a persistent dimerization that likely require this region.

**Fig 7:**
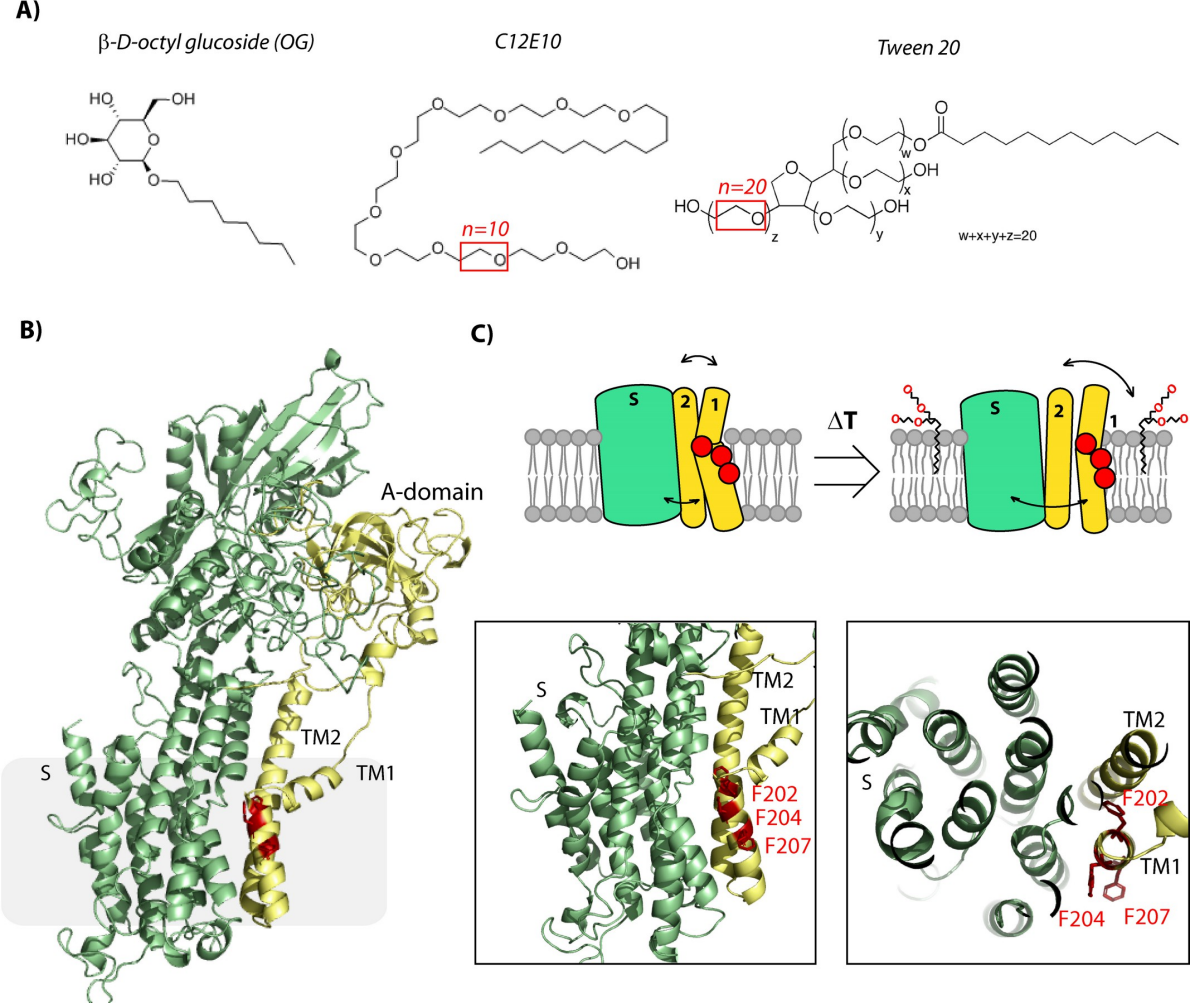
Schematic overview of detergents and Spf1p. A) Detergent structures with the amount of mono glycol ethers in each detergent indicated in red. B) Homology model of the Spf1p structure based on the apo state of the Ca^2+^ ATPase showing location of the conserved phenylalanines in M1 in red, M1-2 and the A domain in yellow and the remainder of the protein (S-domain) in green. Closeup of the M1 helix show location of the phenylalanines which are comparable to the recently published apo structure of Spf1p [13]. C) Schematic overview of the phenylalanines (red dots), M1-2, A-domain (yellow) and M3-10 and S-domain (green) in the absence and presence of polyethylene containing detergents (C12E10/Tween20).

In P-type ATPases transmembrane helixes M1-6 plays an important role in substrate translocation. Initial crystal structures of the Ca^2+^ ATPase showed that transport Ca^2+^ ions are translocated via an open luminal pathway found in this part of the enzyme [32, 33]. Based on detailed analysis of structurally determined intermediate states of the catalytic cycle, the entrance port was later confirmed to be present between the M1/M2 and M3 transmembrane segments [27, 34], where especially M1 appears central in lining the entrance pathway [35]. Notably M1 is highly flexible during transition between the E1 and E2 states, where it in the substrate free E2 state forms a fully kinked α-helix in contrast to being unwound in the substrate bound E1 state. As the M1-M3 transmembrane segments are part of the canonical membrane domain of P-type pumps it is highly likely these helices play a similar role in Spf1p. Indeed, the same entrance pathway was recently shown to be relevant for the P4 lipid flippases [36] which are the closest homologue class of P5 ATPases. The recently presented Cryo-EM structures of Spf1p also show movement of M1 and M2 between the inward open (E1P; AlF_4_-bound) and outward open (E2P; BeF_3_-bound) conformations [13], although the presence of the two additional TM helices in the N-terminal part of Spf1p would conceivably put further restraints on the requirement for coordination between the Ma/Mb and the M1-M3 transmembrane segments during catalytic turnover. It is possible that the detergents identified in the study act by removing this restraint in Spf1p (Fig 7).

Recent reports have made substantial advances towards a function for P5A ATPases in a biological context, but direct biochemical evidence for their transport function and specificity is still missing. Although it is intriguing that P5A type ATPases could act as peptide transporters, or semi-transporters in the case of clearing misfolded TM helixes from the ER membrane, substrate specificity has not yet been studied based on reconstituted biochemical activity. It is our hope that this work will provide an advance for the development of a reconstituted transport assay for a P5A ATPase as have been developed for P5B ATPases [2] and that the data can shed light on the spontaneous nature of activity observed so far. It is proposed that the spontaneous nature of ATP hydrolytic activity could be related to a mechanism of autoregulation that relates to changes the local physicochemical environment of the lipid environment surrounding the transmembrane part of the protein and that this regulation might be lost during purification of the enzyme. Further studies would be needed to prove if this hypothesis is correct.

## Supporting information

S1 Fig Analysis of purification. A-B) A fraction of purified Spf1 was precipitated with TCA and the resulting pellet was subjected to acid hydrolysis after which the individual amino acids where quantified in triplicates using normal phase HPLC (see materials and methods). The corresponding observed values were plotted against the expected values, showing clear linear regression (R^2^ = 0.93) confirming a high degree of purify. C) SDS-PAGE of purified Spf1p. The analysis showed three bands that were cut out and resuspended in a 1:1 mixture of ethanol and 25mM ammonium Bicarbonate and subjected to mass spectrometry (see materials and methods). D) Table of mass spectrometry analysis of the bands using a max quant orbitrap (thermo). Band a. and b. showed mainly Spf1, but band 3 showed a small but significant contamination of glyceraldehyde-3-phosphate dehydrogenase from the cell citric acid cycle. This is a common contaminant of proteins expressed and purified in yeast. The enzyme have no reported no ATPase activity.
(TIF)

S1 Raw images (PDF)
