# Supplementary figures and images for: ATP hydrolytic activity of purified Spf1p correlate with micellar lipid fluidity and is dependent on conserved residues in transmembrane helix M1
Source: PLoS One. 2022 Oct 20;17(10):e0274908. doi: 10.1371/journal.pone.0274908 (PMC9584430; doi:10.1371/journal.pone.0274908)

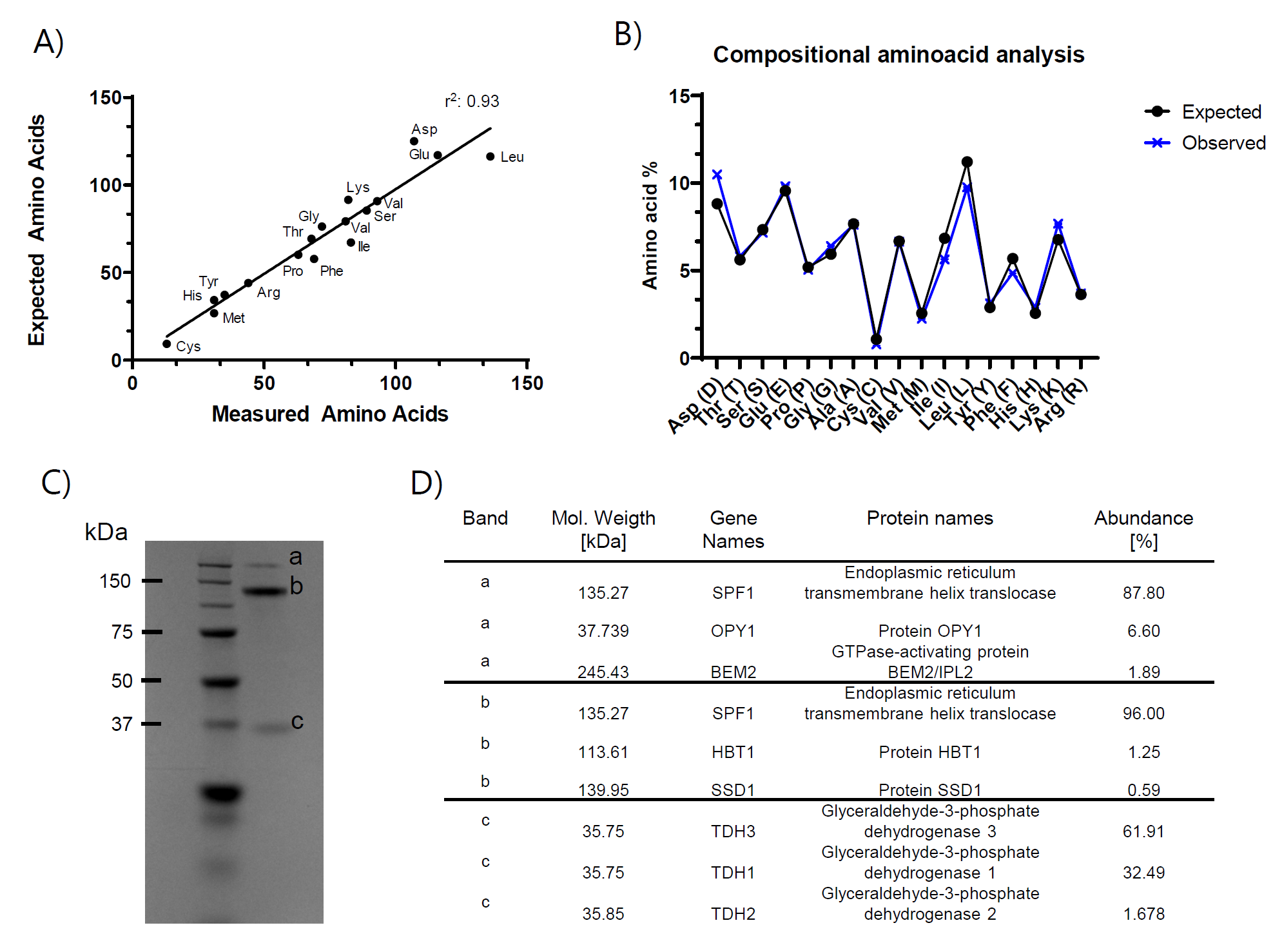

Supplement: S1 Fig — A-B) A fraction of purified Spf1 was precipitated with TCA and the resulting pellet was subjected to acid hydrolysis after which the individual amino acids where quantified in triplicates using normal phase HPLC (see materials and methods). The corresponding observed values were plotted against the expected values, showing clear linear regression (R2 = 0.93) confirming a high degree of purify. C) SDS-PAGE of purified Spf1p. The analysis showed three bands that were cut out and resuspended in a 1:1 mixture of ethanol and 25mM ammonium Bicarbonate and subjected to mass spectrometry (see materials and methods). D) Table of mass spectrometry analysis of the bands using a max quant orbitrap (thermo). Band a. and b. showed mainly Spf1, but band 3 showed a small but significant contamination of glyceraldehyde-3-phosphate dehydrogenase from the cell citric acid cycle. This is a common contaminant of proteins expressed and purified in yeast. The enzyme have no reported no ATPase activity. (TIF) [file pone.0274908.s001.tif]
