# Supplementary material for: ATP hydrolytic activity of purified Spf1p correlate with micellar lipid fluidity and is dependent on conserved residues in transmembrane helix M1
Source: PLoS One. 2022 Oct 20;17(10):e0274908. doi: 10.1371/journal.pone.0274908 (PMC9584430; doi:10.1371/journal.pone.0274908)

Original Uncropped gels for fig 4:

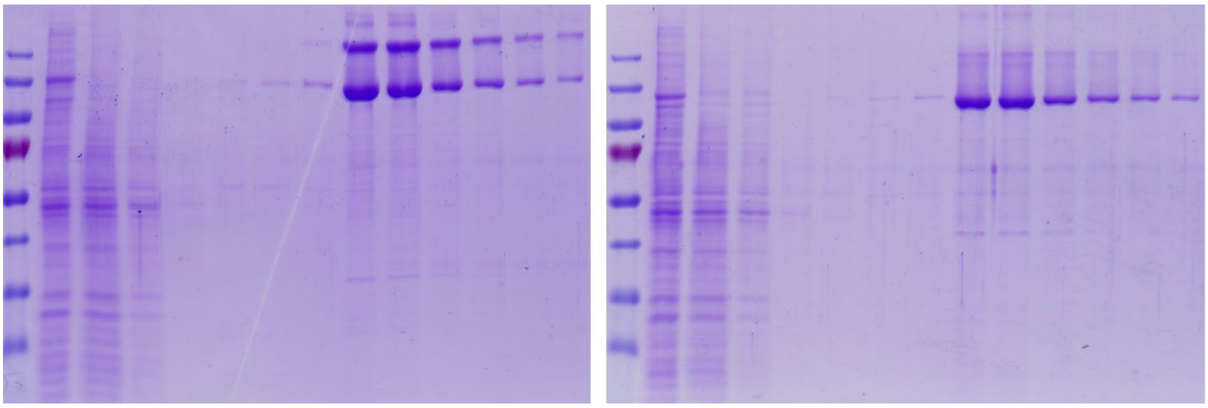

Original Uncropped gels for fig 5E:

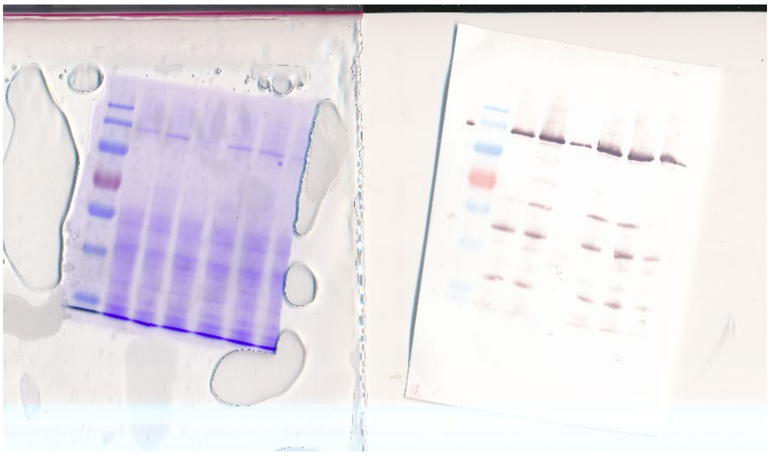

Original Uncropped gels for fig 5F:

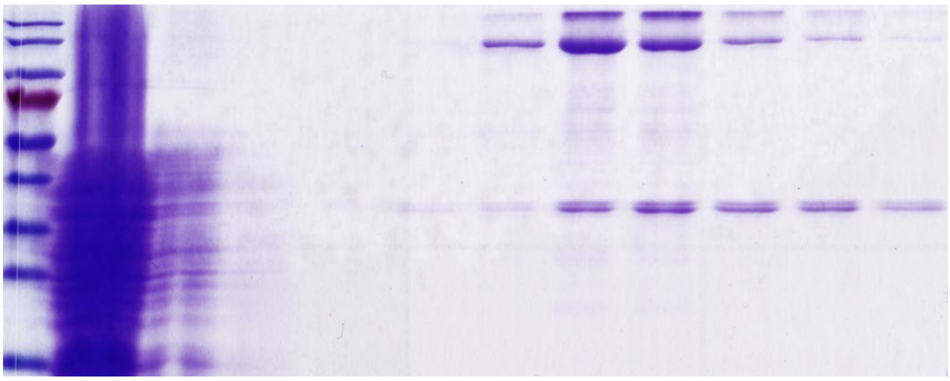

Supplement: S1 Raw images — (PDF) [file pone.0274908.s002.pdf]
